# Morphology, Thermo-Mechanical Properties and Biodegradibility of PCL/PLA Blends Reactively Compatibilized by Different Organic Peroxides

**DOI:** 10.3390/ma14154205

**Published:** 2021-07-28

**Authors:** Marta Przybysz-Romatowska, Mateusz Barczewski, Szymon Mania, Agnieszka Tercjak, Józef Haponiuk, Krzysztof Formela

**Affiliations:** 1 Department of Polymer Technology, Faculty of Chemistry, Gdańsk University of Technology, Gabriela Narutowicza 11/12, 80-233 Gdańsk, Poland; jozef.haponiuk@pg.edu.pl; 2 Institute of Materials Technology, Poznan University of Technology, Piotrowo 3, 61-138 Poznan, Poland; mateusz.barczewski@put.poznan.pl; 3 Department of Chemistry, Technology and Biochemistry of Food, Faculty of Chemistry, Gabriela Narutowicza 11/12, 80-233 Gdańsk, Poland; szymon.mania@pg.edu.pl; 4 Group ‘Materials + Technologies’ (GMT), Department of Chemical and Environmental Engineering, Faculty of Engineering, University of the Basque Country (UPV/EHU), Pza Europa 1, 20018 Donostia-San Sebastian, Gipuzkoa, Spain; agnieszka.tercjaks@ehu.eus

**Keywords:** poly(ε-caprolactone), poly(lactic acid), reactive processing, peroxide initiators, viscoelastic behaviors, thermo-mechanical properties

## Abstract

Reactive blending is a promising approach for the sustainable development of bio-based polymer blends and composites, which currently is gaining more and more attention. In this paper, biodegradable blends based on poly(ε-caprolactone) (PCL) and poly(lactic acid) (PLA) were prepared via reactive blending performed in an internal mixer. The PCL and PLA content varied in a ratio of 70/30 and 55/45. Reactive modification of PCL/PLA via liquid organic peroxides (OP) including 0.5 wt.% of tert-butyl cumyl peroxide (BU), 2,5-dimethyl-2,5-di-(tert-butylperoxy)-hexane (HX), and tert-butyl peroxybenzoate (PB) is reported. The materials were characterized by rotational rheometer, atomic force microscopy (AFM), thermogravimetry (TGA), differential scanning calorimetry (DSC), tensile tests and biodegradability tests. It was found that the application of peroxides improves the miscibility between PCL and PLA resulted in enhanced mechanical properties and more uniform morphology. Moreover, it was observed that the biodegradation rate of PCL/PLA blends reactively compatibilized was lower comparing to unmodified samples and strongly dependent on the blend ratio and peroxide structure. The presented results confirmed that reactive blending supported by organic peroxide is a promising approach for tailoring novel biodegradable polymeric systems with controllable biodegradation rates.

## 1. Introduction

Commercially developed biodegradable aliphatic polyesters, which are still a main focus of academic and industrial research, include both petroleum-based thermoplastics such as poly(ε-caprolactone) (PCL), poly(butyrate succinate) (PBS) and poly(glycolic acid) (PGA), as well as polymers of renewable origins such as poly(lactic acid) (PLA) and polyhydroxyalkanoates (PHA). These materials are continuously popular over the past years due to their potentially hydrolyzable ester bonds, which makes them dominant in the role of biodegradable plastics [1,2,3,4].

Poly(ε-caprolactone) (PCL) is a synthetic biodegradable aliphatic polyester obtained via ring-opening polymerization of ε-caprolactone [5,6]. PCL can be characterized by low melt temperature, usually between 58–60 °C, a low viscosity and its low difficulty of processing [7,8,9]. Despite not being produced from renewable resources, PCL is fully biodegradable when composted [10,11]. The low melting point of PCL makes the material compostable as a rendering medium due to the temperatures obtained during composting routinely exceeding 60 °C [12,13,14].

Poly(lactic acid) (PLA) is a linear aliphatic polyester obtained by polycondensation of naturally produced lactic acid or by catalytic ring-opening of a lactide group [15,16,17,18]. Lactic acid is made via starch fermentation as a by-product of wet corn milling [19,20]. The ester bonds in PLA are sensitive to both chemical hydrolysis and enzymatic chain cleavage [21,22]. Both PCL and PLA are often mixed with starch to increase biodegradability and reduce costs; however, the brittleness of these mixtures is a major disadvantage in many applications [23,24,25].

Research on mixing different polymeric materials together is associated with two main categories. The first is the modification of the properties of the base polymer. Typically, this includes improving mechanical, thermal or processing properties [26,27]. The second category focuses on the need to facilitate, accelerate or delay the biodegradation process. For these reasons, the blending process with other biodegradable polymers appears to be a simple way of modifying blends degradation parameters [28,29].

PCL and PLA are known to be thermodynamically immiscible, which causes their blends to exhibit poor mechanical properties. A lot of research has been considered to improve the miscibility between PCL and PLA [30,31,32,33]. Reactive blending is a well-thought-out method of effective enhancement of miscibility and compatibility of bio-based polymer blends and composites [34]. Dicumyl peroxide (DCP) is the most commonly described and applied peroxide as a free radical producer in reactive compatibilization of bio-based materials. For example, Ji et al. [35] studied the effects of dicumyl peroxide (DCP) on the mechanical properties of PBS/PLA blends. The impact of DCP on the rheological properties of PHB/PLA blends was observed by Dong et al. [36]. They found that the storage modulus and complex viscosity of the blends were increased after the addition of the DCP.

Our previous research also focused on the reactive blending of PCL with PLA [37] or PHB [38] in a weight ratio of 75/25 modified with commercially available and commonly applied organic peroxides, such as di-(2-tert-butyl-peroxyisopropyl)benzene (BIB) or dicumyl peroxide (DCP), used as radical initiator.

This article describes the process of dynamic cross-linking of polymer blends based on biodegradable aliphatic polyesters, including poly(ε-caprolactone) (PCL) and poly(lactic acid) (PLA), which were used in two weight ratios: 70/30 and 55/45, respectively, in the presence of three different types of organic peroxides. The obtained mixtures were subjected to a detailed analysis of their properties in order to obtain new knowledge about the applied method of polymer blend compatibility. The conducted research was carried out to confirm the possibility of achieving compatibility of PCL/PLA blends by using organic peroxides, including tert-butyl cumyl peroxide (BU), 2,5-dimethyl-2,5-di-(tert-butylperoxy)-hexane (HX) and tert-butyl peroxybenzoate (PB). Moreover, the influence of the type of used peroxides on the biodegradation, morphology and final properties, such as mechanical, thermal and rheological properties, of the obtained materials was determined.

## 2. Materials and Methods

### 2.1. Materials

**Figure 1:**
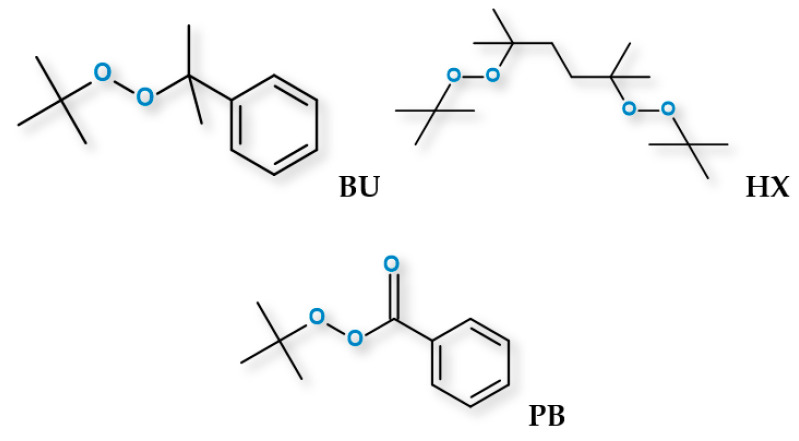
The chemical structures of the organic peroxides.

Poly(ε-caprolactone) (PCL) (Capa 6800) was kindly donated by Perstorp (Malmö, Sweden), while poly(lactic acid) (PLA) grade 3251D was supplied by NatureWorks LLC, (Minnetonka, Minnesota, United States). The characteristics of the used biodegradable polymer are listed in Table 1. The free radical initiators tert-butyl cumyl peroxide (Peroxan BU), 2,5-dimethyl-2,5-di-(tert-butylperoxy)-hexane (Peroxan HX) and tert-butyl peroxybenzoate (Peroxan PB) were supplied by Pergan GmbH (Bocholt, Germany). The chemical structures of the organic peroxides is presented in Figure 1.

### 2.2. Sample Preparation

PCL was blended by reactive mixing with 30 wt.% and 45 wt.% of PLA and further loaded with 0.5 wt.% of various types of organic peroxides, including PB, BU and HX. The samples were prepared in the Brabender^®^ internal mixer (type GMF 106/2) at a screw speed of 100 rpm for a total time of 8 min at 170 °C. First, the PCL and PLA polymers were premixed in a mixing chamber for 4 min, then the reactive compounding with peroxide followed and continued for the next 4 min. Then, the materials were compressed to a 2 mm thickness by the compression molding under a pressure of 4.9 MPa at 170 °C for 1 min and then at room temperature for 5 min. The blends are named PCL*x/y*, where *x* corresponds to the percentage of PCL and *y* corresponds to the type of used organic peroxide. The unmodified PCL/PLA blends were also prepared under the same processing conditions for the properties comparison. The composition and the designation of the studied PCL/PLA blends are described in Table 2.

### 2.3. Methodology

#### 2.3.1. Differential Scanning Calorimetry (DSC)

The thermal properties of the samples were measured by differential scanning calorimetry (DSC) measurement was carried out on a DSC 204 F1 Phoenix apparatus from Netzsch Group (Selb, Germany). The melting point (T_m_), crystallization temperature (T_c_), enthalpy of cold crystallization and enthalpy of melting of each sample were investigated in the temperature range of −80–150 °C under N_2_ atmosphere at a heating rate of 15 °C/min.

The degree of crystallization of the PCL (X_cPCL_) and PLA (X_cPLA_) phase was calculated according to Equation (1):

(1) $$X_{c}\left(\%\right)=\frac{\Delta H_{m}-\Delta H_{\mathrm{cc}}}{\omega \times \Delta H_{0}}\times 100$$

where ΔH_m_ is the specific melting enthalpy, ΔH_cc_ is the enthalpies of cold crystallization, ΔH_0_ is the melting enthalpy of 100% crystalline virgin polymer (where the melting enthalpy of 100% PCL is 136 J/g [39] and 93.7 J/g for PLA [40]), and ω is the weight fraction of the PCL or PLA phase in PCL/PLA blends.

#### 2.3.2. Thermogravimetric Analysis (TGA)

Thermogravimetric analysis was utilized for detecting the thermal degradation process, which is related to the mass loss of the specimen as a function of rising temperature. The analysis of samples was performed using the TG 209F3 apparatus from Netzsch Group (Selb, Germany). Weighted samples (approx. 10 mg) were placed in a corundum dish. The study was carried out in an inert gas atmosphere-nitrogen with a flow rate of 100 mL/min in the range from 25 to 750 °C with a temperature increase rate of 20 °C/min.

#### 2.3.3. Dynamic Mechanical Analysis (DMA)

Dynamic mechanical properties were investigated using a dynamic mechanical analyzer DMA Q800 from TA Instruments (New Castle, Delaware, United States). The PCL/PLA blends in the form of strips (20 × 10 × 2 mm^3^) were measured in single cantilever mode at a constant frequency of 10 Hz as a function of temperature from −100 °C to 80 °C a heating rate of under nitrogen flow.

#### 2.3.4. Mechanical Tests

Static mechanical properties of the PCL/PLA blends, including ultimate tensile strength and elongation at break, were measured in accordance with ISO 527 at room temperature using a Zwick Z020 tensile tester machine provided by ZwickRoell Group (Ulm, Germany) equipped with a 20 kN load cell and a cross-head speed of 10 mm/min. The number of individual samples used in this research was 5 for every tested material.

#### 2.3.5. Rheological Properties

Rheological properties of the PCL/PLA blends were tested using a rotational rheometer Anton Paar MCR 301 (Graz, Austria) equipped with a parallel-plate fixture (25 mm diameter) in oscillation shearing mode at a temperature of 170 °C. During dynamic oscillatory measurements, for all the samples’ strain amplitude sweep experiments were performed at 170 °C with a constant angular frequency of 10 Hz in the varying strain window 0.01%–100%. Based on the amplitude sweep tests, the 2% strain was selected for frequency sweep experiments. For all considered materials selected value is in the range of linear viscoelastic region (LVE). The frequency sweep measurements were realized in the 0.05–100 rad/s angular frequency (ω) range.

#### 2.3.6. Atomic Forace Microscopy (AFM)

Atomic Force Microscopy images were obtained with a Nanoscope V scanning probe microscope Multimode 8 Digital Instruments from Bruker (Billerica, Massachusetts, United States) under ambient conditions. Tapping Mode (TM) was employed in the air using an integrated tip/cantilever (125 μm in length with ca. 300 kHz resonant frequency). Typical scan rates during recording were 0.7 to 1 line/s using a scan head with the maximum range of 50 μm.

#### 2.3.7. Biodegradability Tests

The biodegradability of the PCL/PLA blend was assessed with a standard method (OECD 301 F) involving the measurement of oxygen consumption by microorganisms using the OxiTop-IDS A 12 equipment from WTW (Wrocław, Poland). Activated sludge, mainly municipal with 30 mg/L dry mass, from sewage treatment plant (Saur Neptun Gdansk S.A., Gdansk, Poland) was used as the inoculum. The standard sample volume was 164 mL. This enabled the determination of BOD values up to 400 mg/L in the water phase. The solids are weighed directly into the reactors in the amount of approx. 16.4 mg/bottle, after prior mechanical milling of the PCL/PLA samples using laboratory mill FW 135 from ChemLand (Stargard, Poland). Then, sludge suspension and 3 drops of nitrification inhibitor NT600 were poured into each bottle, where a magnetic stir bar was immersed. A rubber carrier with a carbon dioxide absorber (0.4 g NaOH) was then placed in each bottle. Finally, OxiTop measuring heads were fixed, sealed bottles were placed on a stirring platform and put into a thermostatic cabinet. The incubation was conducted for 28 days at 37 ± 1 °C, during which the OxiTop Controller collected the measured values every 24 h. Three measurements of the biodegradability of the obtained blends were taken. In each test series, blank and reference tests (sodium benzoate with a concentration of 100 mg/L) were performed in parallel.

## 3. Results and Discussion

### 3.1. DSC of Organic Peroxides and Their Influence on PCL/PLA Blends Thermal Properties

**Figure 2:**
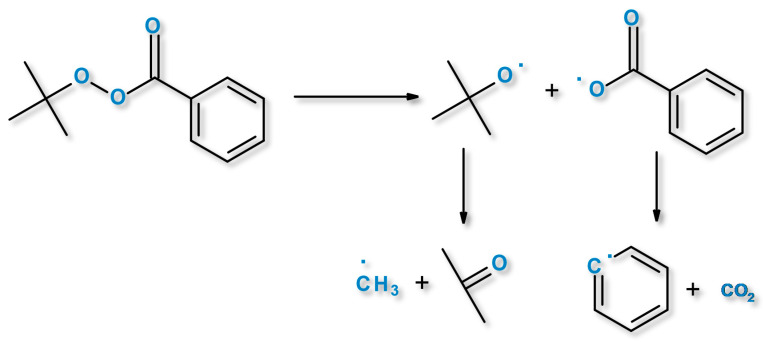
A scheme of the thermal decomposition mechanism of PB.

The organic peroxides can have low overall hydrogen abstraction ability, which causes less cross-linking due to their lower free radical efficiency. This is related to the hydrogen abstraction ability of derived radicals formed during peroxide decomposition. The decomposition of the organic peroxides gives primary radicals that can be a source for secondary alkyl or aryl, which are formed by β-scission of primary radicals. Example decomposition mechanism of PB peroxide is shown in Figure 2, where PB gives rise to benzoyloxy and t-butoxy radicals.

**Figure 3:**
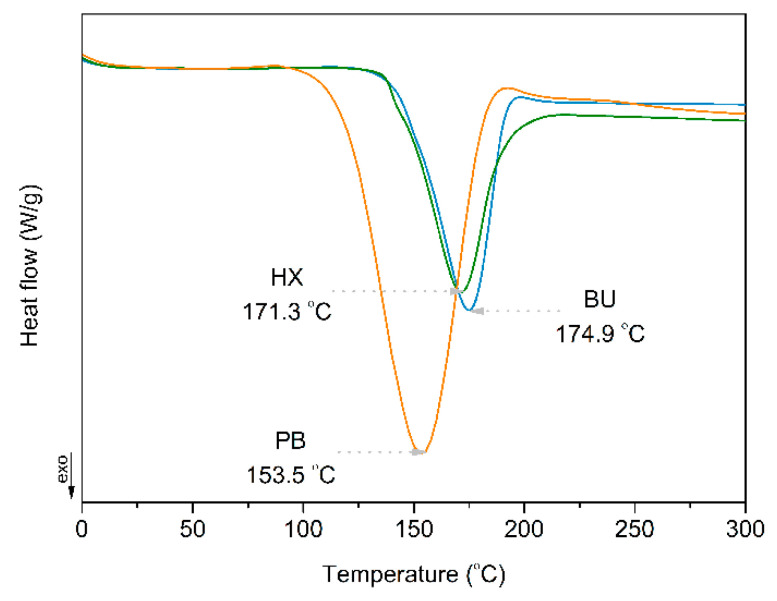
Heat flow vs. temperature for the thermal decomposition of BU, HX and PB peroxides.

DSC curves of three types of peroxides are shown in Figure 3. The decomposition temperature (T_decomp._) was determined at the first heating scan. The main parameter T_decomp._ was equal to 174.9 °C for BU, and for HX, it was similar, equaling 171.3 °C. In the case of PB peroxide, the temperature parameter was significantly lower at 153.5 °C, respectively. The results of DSC analysis allowed defining temperature of processing during the blending of PCL/PLA system modified via free radical reaction initiated of organic peroxide.

**Figure 4:**
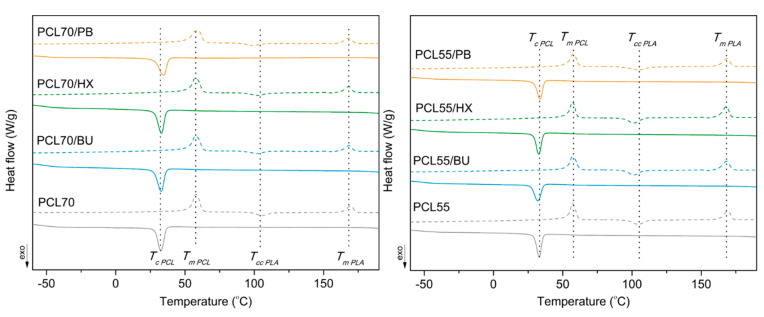
DSC curves of the second heating for PCL/PLA blends.

Figure 4 shows the results of the DSC analysis that was carried out to understand the crystallization and melting behavior of PCL/PLA blends. The detailed data of thermal parameters are listed in Table 3, including the melting temperature (T_m PCL_), melting enthalpy (ΔH_m PCL_), crystallization temperature (T_c PCL_), crystallization enthalpy (ΔH_c PCL_) and the degree of crystallinity (X_c PCL_) of PCL, as well as melting temperature (T_m PLA_), melting enthalpy (ΔH_m PLA_) and cold crystallization temperature (T_cc PLA_) of PLA. These temperatures are also indicated on the *X*-axis of Figure 4. The T_m_, T_c_ and X_c_ of unmodified PCL/PLA blends were 57.8 °C, 32.7 °C and 40.7%, respectively, for the 70/30 ratio. The T_m_, T_c_ and X_c_ of unmodified PCL/PLA blends were 57.1 °C, 29.9 °C and 39.3%, respectively, for the 55/45 ratio. These mentioned parameters have not changed significantly for PCL/PLA blends modified by organic peroxides. Furthermore, the melting point of PCL is overlapped with a glass transition of PLA, and it is not visible. The T_m_ of PLA shifted towards a lower temperature for the PCL/PLA blends modified by peroxides, as was observed. The aforementioned parameter of decomposition temperature of organic peroxides was ranging between approximately 150 °C and 175 °C. Therefore, it cannot be visible on the DSC curves of the studied blends, which confirms that the peroxides decomposition process, which occurs during the reactive blending, simultaneously causes the cross-linking of PCL, PLA and both phases.

### 3.2. Thermal Stability of PCL/PLA Blends

**Figure 5:**
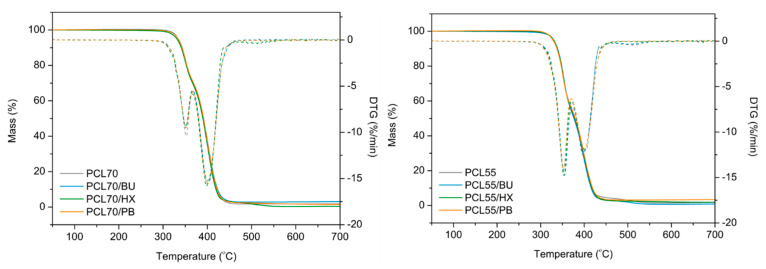
TGA and DTG curves of PCL/PLA blends.

The thermal stability of the PCL/PLA blends was investigated by thermogravimetric analysis (TGA) and derivative thermogravimetry (DTG). The obtained results are presented in Figure 5. DTG curves showed that the thermal degradation of blends occurs in two zones. The first step is related to PLA decomposition, and the second one is associated with the PCL degradation. It is obvious that the higher the content of PLA in the blends, the thermal stability decreases, which can be related to the lower degradation temperature of PLA in comparison to PCL [41]. The temperatures corresponding to the −2%, −5%, −10% and −50% mass loss for PCL/PLA blends are essential for evaluating their thermal stability and are summarized in Table 4, which show that the apparent thermal stability of the PCL/PLA blend does not change significantly after modification by the organic peroxides. Major differences are visible for the T-2% parameter and char residue, which can be related to the efficiency of cross-linking for each peroxide and formulation of volatile by-products during peroxide decomposition (see Figure 2). However, the results of TGA do not indicate which of the samples is most thermally stable because for each composition of PCL/PLA blends, the changes are minimal.

### 3.3. Dynamic Mechanical Analysis

**Figure 6:**
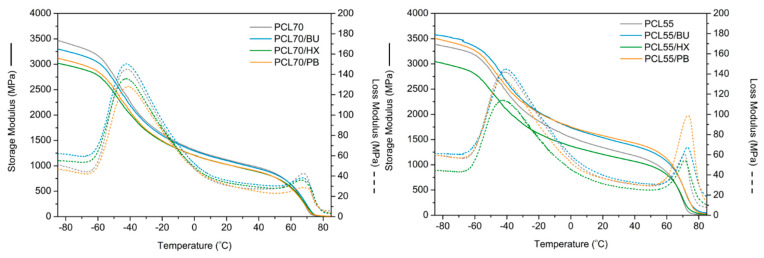
Storage and loss modulus for PCL/PLA blends (70/30) and (55/45).

Dynamic mechanical analysis (DMA) results for the blends with various PLA contents are presented in Figure 6, which shows the temperature dependence of storage and loss modulus curves of PCL/PLA blends at various compositions. Based on the DMA results, it can be noticed that blending PCL with PLA at a proportion of 55/45 caused an interruption in PLA crystallization, which is observed in the storage modulus decreasing, as compared to unmodified PCL/PLA in the weight ratio of 70/30. The same conclusion can be made from the DSC analysis (X_PLA_) described in Section 3.1. The PCL/PLA blends exhibited two peaks at −42 °C and 70 °C that were attributed to the glass transition temperature (T_g_) of PCL and PLA, respectively. The T_g_ (70 °C) of PLA in unmodified PCL/PLA blend was slightly higher than the T_g_ (65 °C) of PLA in the PLA/PCL blends modified by PB, indicating that there was some better molecular interaction between the two components due to the cross-linking or branching reaction.

### 3.4. Mechanical Tests

**Figure 7:**
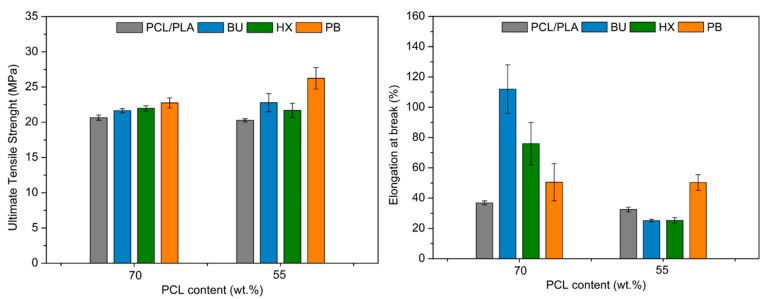
Ultimate tensile strength and elongation at break of PCL/PLA blends modified by OP. Graphs show the differences in mechanical properties as function th PCL content in the blends.

The results of the ultimate tensile strength and elongation at break of studied PCL/PLA blends are shown in Figure 7. It was found that after modification by organic peroxides, the ultimate tensile strength and elongation at break for PCL/PLA blends in almost every weight ratio in the presence of peroxides obviously increased. In this case, when the weight ratio equals 55/45, only the addition of PB positively affects the elongation at break of the PCL/PLA blends, while the peroxides, such as BU or HX, decreased the elongation at break. It confirms that the free radicals enhance the interaction between PCL and PLA. The blends with more content of PLA provide higher tensile strength but have a lower elongation at break. It is associated with PLA properties, which are brittle and have a low elongation at break. The elongation at break value of PCL/PLA in the weight ratio of 70/30 modified with all peroxides shows significant improvement, which can be advance during polymeric film formulation. This is especially visible for BU peroxide, suggesting that this peroxide caused the occurrence of the PCL–PLA copolymer and therefore the improvement of compatibilization and miscibility of this blend. The addition of PB, however, yields different results, which indicate that cross-linking is occurring. These conclusions are also confirmed by the biodegradability test in the following section.

### 3.5. Rheological Study

**Figure 8:**
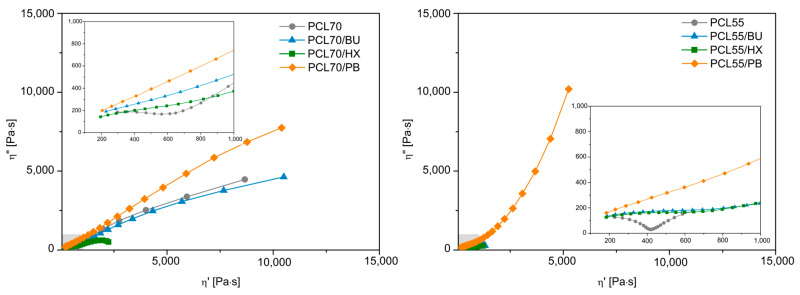
Cole–Cole plots for PCL/PLA blends at various weight ratios ((70/30) and (55/45)) and modified by OP.

The rheological parameters measured at 170 °C, including storage modulus (G′), loss modulus (G″) and complex viscosity (η*), were very sensitive to the morphology development of the PLA/PCL immiscible mixture and various types of organic peroxide modified blends. The presence of morphological changes in the PCL/PLA blends can also be revealed unequivocally with Cole–Cole plots (Figure 8), which are based on a presentation of imaginary (η″) and real (η′) viscosity parts of a complex viscosity. This plot is commonly applied to describe the viscoelastic properties of materials that have a high relaxation time distribution, highlighting the phase separation changes. As shown in Figure 8, an apparition of a second semi-circular arc is present only for the unmodified PCL/PLA composition, highlighting the immiscibility of the blends at blending ratios. The addition of 0.5 wt % of OP in the PCL/PLA blends highlights a completely different behavior. The OP makes a huge impact on the homogeneity of the blends, as only one relaxation is obtained on the full range of ratios, which can be observed in the plots. This clearly indicates long-chain branching, cross-linking, and grafting of PCL and PLA, caused by free radicals from peroxides, which act as compatibilizers. While the overall thermo-rheological miscibility of PCL-PLA blends is still limited, the incorporation of OP allows to partially reduce the phase separation of the polymers in a molten state. It should be mentioned that only for the 55/45 blend modified by PB, the significant upward inflection of the η″(η′) curve was observed. This phenomenon is usually related to yield behavior [42], which, in the presented case, may result from cross-linking of the blend structure. All of these important observations are an indication of the improvement of the miscibility between PCL and PLA due to the reactive modification. OP plays a major role in giving remarkable rheological property improvement due to grafting.

**Figure 9:**
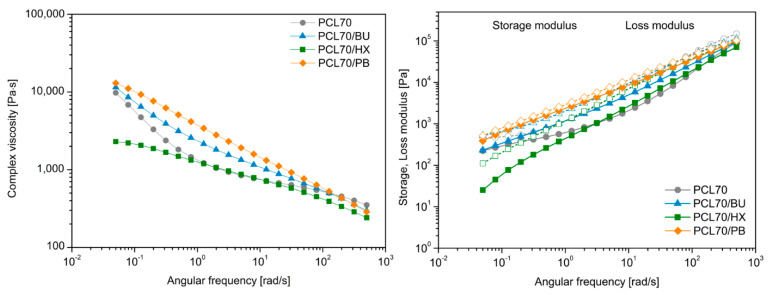
Complex viscosity, storage and loss modulus of unmodified PCL/PLA blends with weight ratio of 70/30 and modified by OP.

**Figure 10:**
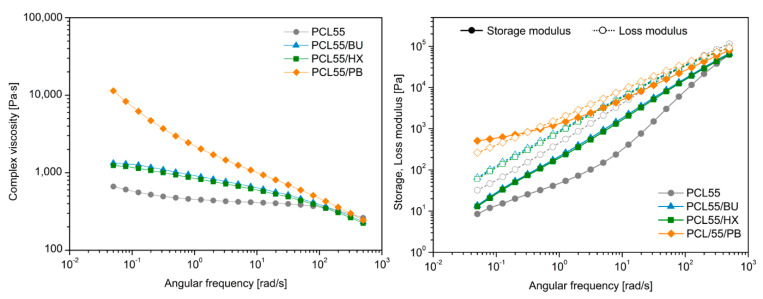
Complex viscosity, storage and loss modulus of unmodified PCL/PLA blends with weight ratio of 55/45 and modified by OP.

To provide more information regarding the interactions between immiscible polymers, angular frequency tests were performed on all samples, both unmodified and modified by OP. The logarithm of the dynamic modulus and the complex viscosity as a function of the logarithm of angular frequencies was measured at 170 °C and is shown for PCL/PLA blends with the weight ratios of 70/30 and 55/45 in Figure 9 and Figure 10, respectively. An overall observation is that the dynamic storage modulus and loss modulus of all the blends increases as the frequency increases, which is characteristic of a viscoelastic liquid. For all of the PCL/PLA blends modified by BU, HX and PB, although the enhancement in loss modulus is insignificant, there is a great increase in the storage modulus. The complex viscosity versus frequency results showed that PCL exhibited typical Newtonian fluid behavior with the invariant of the complex viscosity in a wide range of frequencies. The complex viscosity of the modified PCL/PLA (55/45) was significantly higher, especially for PCL55/PB, than the unmodified PCL/PLA (55/45) at low frequencies; some researchers attributed this phenomenon to a formation of network-like structures [42]. Moreover, the creation of physical or chemical 3D structures is often observed in the case of thermoplastics as a limited dependency of G′ by ω at the low-frequency range [43,44], which is considered case is also noted only for 55/45/PB series.

### 3.6. Morphology

**Figure 11:**
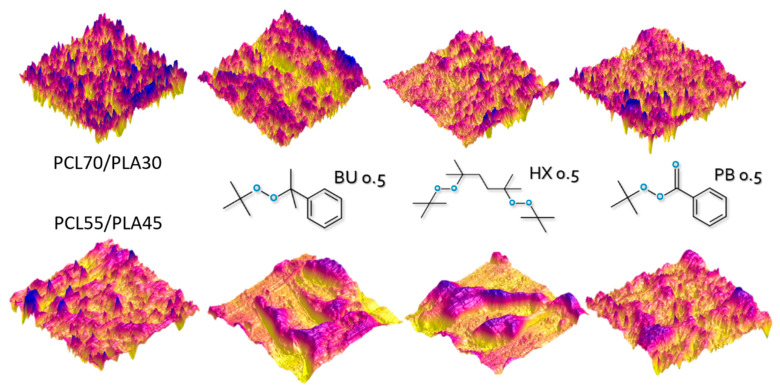
The effect of different types of organic peroxide on the morphology of PCL/PLA blends in the weight ratio of 55/45.

Conventional AFM topography imaging in tapping mode was conducted on the PCL/PLA blends in the weight ratio of 55/45 with resulting topographic and phase images. The 50 μm × 50 μm 3D image in Figure 11 reveals a PCL matrix containing PLA domains for unmodified PCL/PLA blend. The PLA domains can be distinguished by their slightly rougher surface and greater height (dark contrast) in the topographic image. The application of low molecular weight additives, such as organic peroxides, leads to a smooth surface, especially for PCL/PLA (55/45) modified by PB. Moreover, the morphology of PCL/PLA (55/45) blends modified by BU or HX suggests its co-continuity.

### 3.7. Biodegradability

In order to investigate biodegradation efficiency of PCL/PLA blends the OxiTop technique was applied. It is well known that oxygen consumption is strongly correlated with the respiratory activity of microorganisms. During the analysis, the air pressure is measured after trapping the produced carbon dioxide into a strong base solutions monitoring. This technique started to be popular because it allows to observe the transformation of organic matter in a particular environment [45]. However, literature data indicate that so far not many studies have been conducted on the biodegradation of biodegradable polymers (e.g., PLA) under aquatic conditions compared to the widely investigated terrestrial systems [46,47,48]. The results of biodegradation of PCL/PLA blends are presented in the Table 5. Results shown that whatever the PCL/PLA blend used, there is a consumption of O_2_, thus confirming the multiplication and growth of aerobic bacteria and their ability to assimilate prepared PCL/PLA blends as only carbon source.

## 4. Conclusions

Poly(ε-caprolactone)/poly(lactic acid) (PCL/PLA) blends are promising materials with biodegradable properties and adjustable performance for many applications. According to the results obtained, blending PCL with higher content of PLA caused a decrease in thermal properties of the blend with little to no impact on the mechanical properties. However, the addition of organic peroxides, while it did not impact the thermal properties, positively influenced the mechanical properties. On the basis of the data obtained, selected peroxides are able to improve the miscibility between PCL and PLA, which is indicated by the results of rheological and morphology analyses, as well as the enhancement of the mechanical properties. For all the tested samples, the proportion of biodegradable material increased with the duration of the test. The use of peroxides during the reactive extrusion process significantly reduced the biodegradation of materials, which results from their cross-linking. This limitation was the result of the mass ratio of PCL and PLA in the composite, the type of peroxide used and its concentration in the mixture. Although the addition of peroxides slowed down the biodegradation process, it did not stop it completely, which makes them advantageous and promising additives for achieving cross-linking and/or compatibilization of PCL and PLA.

## Figures and Tables

**Table 1:** The characteristics of the used materials.

Parameter	PCL 6800	PLA 3251D
Density, g/cm^3^	1.15	1.24
Melting point, °C	58–60	155–170
Glass transition (T_g_), °C	−60	55–60
Average molecular weight (M_w_), g/mol	80,000	55,400
Melt flow index (MFI)_190 °C/2.16kg_, g/10 min	4.1	35.0

**Table 2:** The composition and the designation of the studied PCL/PLA blends.

Sample Name	Polymers (wt. %)		Organic Peroxides (wt. %)		
	PCL	PLA	BU	HX	BP
PCL70	70	30	-	-	-
PCL70/BU	70	30	0.5	-	-
PCL70/HX	70	30	-	0.5	-
PCL70/PB	70	30	-	-	0.5
PCL55	55	45	-	-	-
PCL55/BU	55	45	0.5	-	-
PCL55/HX	55	45	-	0.5	-
PCL55/PB	55	45	-	-	0.5

**Table 3:** DSC parameters (2nd heating) of PCL/PLA blends and PCL/PLA modified by OP.

Sample Code	*T*_m PCL_ (°C)	Δ*H*_m PCL_ (J/g)	*T*_c PCL_ (°C)	Δ*H*_c PCL_ (J/g)	*T*_m PLA_ (°C)	Δ*H*_m PLA_ (J/g)	*T*_cc PLA_ (°C)	Δ*H*_cc PLA_ (J/g)	X_c PCL_ (%)	X_c PLA_ (%)
PCL70	57.8	38.7	32.7	42.9	169.5	15.3	105.6	9.6	40.7	20.3
PCL70/BU	57.6	36.4	32.8	42.9	168.3	13.9	102.0	8.6	38.2	18.9
PCL70/HX	57.5	36.7	32.9	42.7	168.1	14.0	102.5	8.7	38.4	18.9
PCL70/PB	58.1	37.7	34.4	40.9	168.3	14.1	98.5	7.2	39.6	24.5
PCL55	57.1	29.4	32.9	34.0	169.2	19.8	104.4	13.4	39.3	15.2
PCL55/BU	57.2	29.8	32.0	32.8	168.1	22.4	101.2	13.8	39.8	20.4
PCL55/HX	57.0	27.6	32.7	32.9	168.2	24.6	101.2	14.9	36.9	23.0
PCL55/PB	57.7	27.9	33.6	31.5	168.6	19.6	104.7	13.3	37.3	14.9

**Table 4:** Thermal degradation parameters of PCL/PLA blends.

Sample Codes	T_−2%_ (°C)	T_−5%_ (°C)	T_−10%_ (°C)	T_−50%_ (°C)	R_716.5 °C_ (%)
PCL70	324.0	334.3	343.1	391.2	1.87
PCL70/BU	324.6	333.9	342.5	391.6	3.13
PCL70/HX	317.6	331.2	341.0	389.9	0.44
PCL70/PB	322.8	333.9	342.8	391.4	1.50
PCL55	317.5	329.5	338.4	379.5	1.89
PCL55/BU	316.8	329.4	338.7	376.5	0.94
PCL55/HX	318.7	329.4	338.4	378.1	1.92
PCL55/PB	317.9	328.1	337.3	379.4	3.50

**Table 5:** The results of the biodegradation of PCL/PLA blends.

Sample Codes	Biodegradability [% TOD] After		
	7 Days	14 Days	28 Days
PCL70	1.87 ± 0.10	7.24 ± 0.26	21.04 ± 0.52
PCL70/BU	0.70 ± 0.04	4.18 ± 0.10	7.23 ± 0.16
PCL70/HX	0.23 ± 0.01	1.79 ± 0.04	4.04 ± 0.12
PCL70/PB	0.31 ± 0.01	2.03 ± 0.05	4.04 ± 0.09
PCL55	1.22 ± 0.04	4.73 ± 0.12	15.93 ± 0.16
PCL55/BU	0.39 ± 0.02	2.13 ± 0.09	9.37 ± 0.19
PCL55/HX	0.10 ± 0.01	1.05 ± 0.05	3.77 ± 0.20
PCL55/PB	0.16 ± 0.02	1.76 ± 0.07	6.78 ± 0.15

## Data Availability

Not applicable.
